# Predicting lymph node metastasis in papillary thyroid carcinoma: radiomics using two types of ultrasound elastography

**DOI:** 10.1186/s40644-025-00832-w

**Published:** 2025-02-13

**Authors:** Xian-Ya Zhang, Di Zhang, Wang Zhou, Zhi-Yuan Wang, Chao-Xue Zhang, Jin Li, Liang Wang, Xin-Wu Cui

**Affiliations:** 1 Department of Medical Ultrasound, Tongji Hospital, Tongji Medical College, Huazhong University of Science and Technology, No. 1095, Jiefang Avenue, Wuhan, Hubei Province 430030 China; 2 Department of Medical Ultrasound, The First Affiliated Hospital of Anhui Medical University, Hefei, 230022 China; 3 Department of Medical Ultrasound, The Affiliated Cancer Hospital of Xiangya School of Medicine, Central South University, Changsha, 410031 China; 4 Computer Center, Tongji Hospital, Tongji Medical College, Huazhong University of Science and Technology, No. 1095, Jiefang Avenue, Wuhan, Hubei Province 430030 China

**Keywords:** Radiomics, Ultrasound elastography, Papillary thyroid carcinoma, Lymph node metastasis, Peritumoral region

## Abstract

**Background:**

To develop a model based on intra- and peritumoral radiomics features derived from B-mode ultrasound (BMUS), strain elastography (SE), and shear wave elastography (SWE) for cervical lymph node metastasis (LNM) prediction in papillary thyroid cancer (PTC) and to determine the optimal peritumoral size.

**Methods:**

PTC Patients were enrolled from two medical centers. Radiomics features were extracted from intratumoral and four peritumoral regions with widths of 0.5–2.0 mm on tri-modality ultrasound (US) images. Boruta algorithm and XGBoost classifier were used for features selection and radiomics signature (RS) construction, respectively. A hybrid model combining the optimal RS with the highest AUC and clinical characteristics as well as a clinical model were built via multivariate logistic regression analysis. The performance of the established models was evaluated by discrimination, calibration, and clinical utility. DeLong’s test was used for performance comparison. The diagnostic augmentation of two radiologists with hybrid model’s assistance was also evaluated.

**Results:**

A total of 660 patients (mean age, 41 years ± 12 [SD]; 506 women) were divided into training, internal test and external test cohorts. The multi-modality RS_1.0 mm_ yielded the optimal AUCs of 0.862, 0.798 and 0.789 across the three cohorts, outperforming other single-modality RSs and intratumoral RS. The AUCs of the hybrid model integrating multi-modality RS_1.0 mm_, age, gender, tumor size and microcalcification were 0.883, 0.873 and 0.841, respectively, which were significantly superior to other RSs and clinical model (all *p* < 0.05). The hybrid model assisted to significantly improve the sensitivities of junior and senior radiologists by 19.7% and 18.3%, respectively (all *p* < 0.05).

**Conclusions:**

The intra-peritumoral radiomics model based on tri-modality US imaging holds promise for improving risk stratification and guiding treatment strategies in PTC.

**Trial registration:**

Retrospectively registered.

**Supplementary Information:**

The online version contains supplementary material available at 10.1186/s40644-025-00832-w.

## Background

Thyroid cancer ranks as the 7th most common in cancer incidence [1]. Papillary thyroid cancer (PTC), as a main contributing factor, accounts for 89.1% of overall thyroid cancer [2]. Approximately 30–80% of PTC patients harbor cervical lymph node metastasis (LNM) [3], which has been documented to be well relevant to an increased risk of local recurrence, distant metastasis, and mortality [4]. According to the 2015 American Thyroid Association (ATA) guideline, therapeutic lymph node dissection (LND) is recommended for patients with clinical or imaging evidence of cervical LNM [5]. Yet the consensus about whether prophylactic LND is mandatory for clinically node-negative (cN0) PTC patients remains contentious [6]. Unnecessary prophylactic LND may increase the risk of nerve injury, vocal cord palsy, and hypoparathyroidism [5, 7]. Consequently, the accurate preoperative LNM evaluation holds paramount significance and further provides a basis for subsequent treatment protocol formulation.

Ultrasound (US) has been suggested for all malignant or suspected malignant thyroid nodules for preoperative evaluation of cervical lymph node (LN) [5]. Despite advancements in imaging and diagnostic technologies, accurately predicting LNM remains challenging. It has been reported that the limited sensitivity of US for diagnosing LNM is 51% [8]. The sensitivity of US for central compartment is even lower, at only 28% [9]. Ultrasound elastography (USE) is recommended as an additional imaging technique to B-mode US (BMUS) for measuring tissue stiffness, which mainly includes strain elastography (SE) and shear wave elastography (SWE) [10]. SE depicts relative stiffness by estimating the tissue displacements induced by manual compression or physiologic shifts [11]. SWE quantitatively provides the tissue stiffness by measuring the speed (in m/s) of shear waves produced by acoustic radiation force impulse [11]. Previous studies have indicated the value of SE or SWE in predicting cervical LNM in PTC patients as tumor stiffness increased with tumor invasion [12–14]. Additionally, cancer cells tend to infiltrate into surrounding tissues as disease progresses, resulting in higher stiffness of peritumoral tissues [15]. Several studies suggested that peritumoral stiffness might have clinical association with tumor biological behavior [16–18]. However, the potential of surrounding stiffness of thyroid tumor for cervical LNM estimation has yet to be investigated.

Radiomics offers the superiority of extracting substantially more features than visual imaging analysis, providing high-dimensional quantitative features that characterize tumor heterogeneity, phenotype, and microenvironment [19]. Numerous studies indicated that radiomics could be used as a triage tool for LNM prediction [20–22]. However, most previous studies focused on the internal radiomics features within the tumor with no consideration of the variations induced by lymphatic metastasis in the peritumoral tissue. Primary tumor metastasizes to regional LNs via functional lymphatics within peritumoral tissue, which has biological importance in the LNM process [23, 24]. Recent studies have confirmed that radiomics analysis encompassing both intra- and peritumoral regions significantly enhanced diagnostic accuracy compared to intratumoral region alone [25, 26]. However, the potential value of peritumoral radiomics in predicting LNM in PTC as well as the optimal width of peritumoral region has not been definitively explored. Few studies have systematically evaluated the predictive value of specific peritumoral regions at defined distances.

Multimodal radiomics have shown promise in improving diagnostic accuracy. However, existing studies often neglect the contribution of specific peritumoral regions and fail to fully integrate novel imaging modalities such as USE. Therefore, this study aims to address these gaps by systematically evaluating the diagnostic value of peritumoral radiomics features across various margins and integrating multimodal US with clinical data and to determine the optimal peritumoral region width for LNM prediction in PTC. The comprehensive of peritumoral elasticity indices for LNM prediction will also be explored.

## Methods

### Study population

The study was approved by the Institutional Review Board (approval number: 2023S129). The requirement for informed consent was waived for the retrospective study. The computer code used for modeling data analysis are available at https://github.com/nywl/peritumor_zxy.git.

**Fig. 1:**
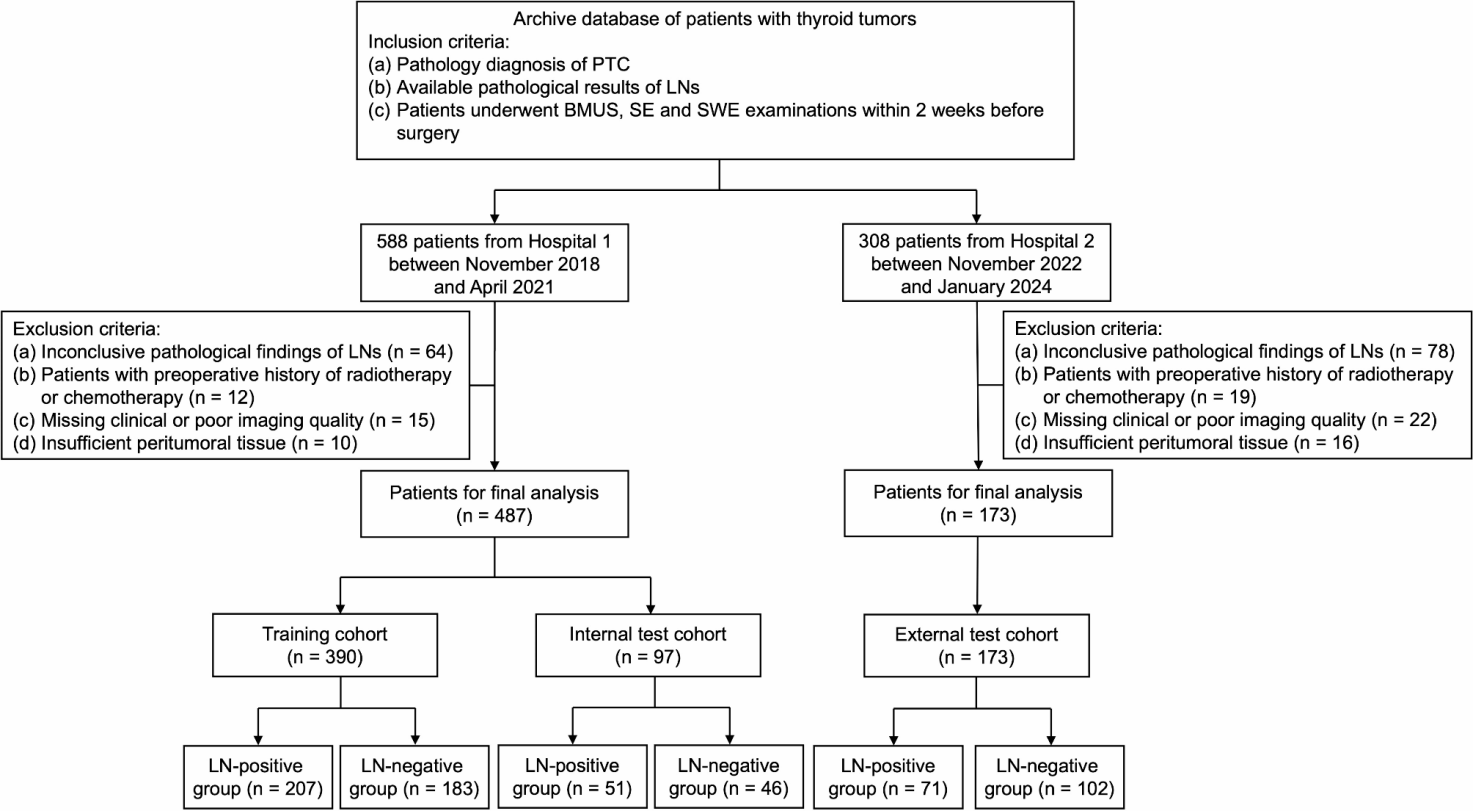
The flowchart of patient enrollment. BMUS: B-mode ultrasound; LN: lymph node; PTC: papillary thyroid carcinoma; SE: strain elastography; SWE: shear wave elastography

The inclusion criteria were as follows: (1) tumor was detected by US or other imaging modality and pathologically identified as PTC; (2) LN status was confirmed by LND; (3) multi-modality US examinations, including BMUS, SE and SWE, were conducted within 2 weeks before surgery; (4) peritumoral normal tissue at the same depth and US cross-section was adequate. The exclusion criteria included: (1) pathological findings of cervical LNs were inconclusive; (2) patients with preoperative history of radiotherapy or chemotherapy; (3) clinical information was missing or imaging quality was poor; (4) peritumoral tissue was not sufficient for analysis. Figure 1 displays the process for patient recruitment. The data on baseline clinical characteristics were derived from the electronic patient record, including age, gender, nodule pathology and cervical LN status.

### Multimodal ultrasound imaging protocol and image analysis

The BMUS, SE and SWE examinations of each target thyroid tumor were performed using Resona 7 and Resona DC-8 US system (Mindray Medical International, Shenzhen, China) with a line array probe (L14-5 W) at a frequency of 4.0–14.0 MHz by board-certified radiologists with 10 years of experience in thyroid US and 5 years of experience in USE within the two centers. For patients with multiple tumors, the tumor with the largest diameter was selected as the target tumor.

Conventional US examination was first conducted. The BMUS characteristics were analyzed and the American College of Radiology Thyroid Imaging Reporting and Data System (ACR TIRADS) category was assigned when images were acquired [27]. The BMUS characteristics of target tumor included size, primary site, composition, echogenicity, margin, shape and microcalcification.

Subsequent SE and SWE examinations were performed by the same radiologist at the same plane with the patient’s position remaining unchanged. Elasticity score (ES) and strain ratio (SR) were identified for SE evaluation. The ES was determined by the radiologist using the five-point scoring system [15]. For SR calculation, the B/A, B/shell_0.5_, B/shell_1.0,_ B/shell_1.5_ and B/shell_2.0_ were calculated automatically, representing the SR of the internal tumor and that of the peritumoral area from 0.5 to 2 mm width, respectively. For SWE indices measurement, the maximum, minimum, mean and standard deviation (SD) of elasticity values (kPa) of the tumor and the surrounding tissue with the widths of 0.5–2 mm were automatically calculated. Additionally, the qualitative feature named “stiff rim” sign, was evaluated in SWE, which was recognized as the typically increased peritumoral stiffness and coded in orange or red [28]. To improve data reproducibility, three measurements were conducted and the average of each variable was calculated. Detailed imaging protocol is presented in the Appendix A.1.

### ROI segmentation and radiomics feature extraction

**Fig. 2:**
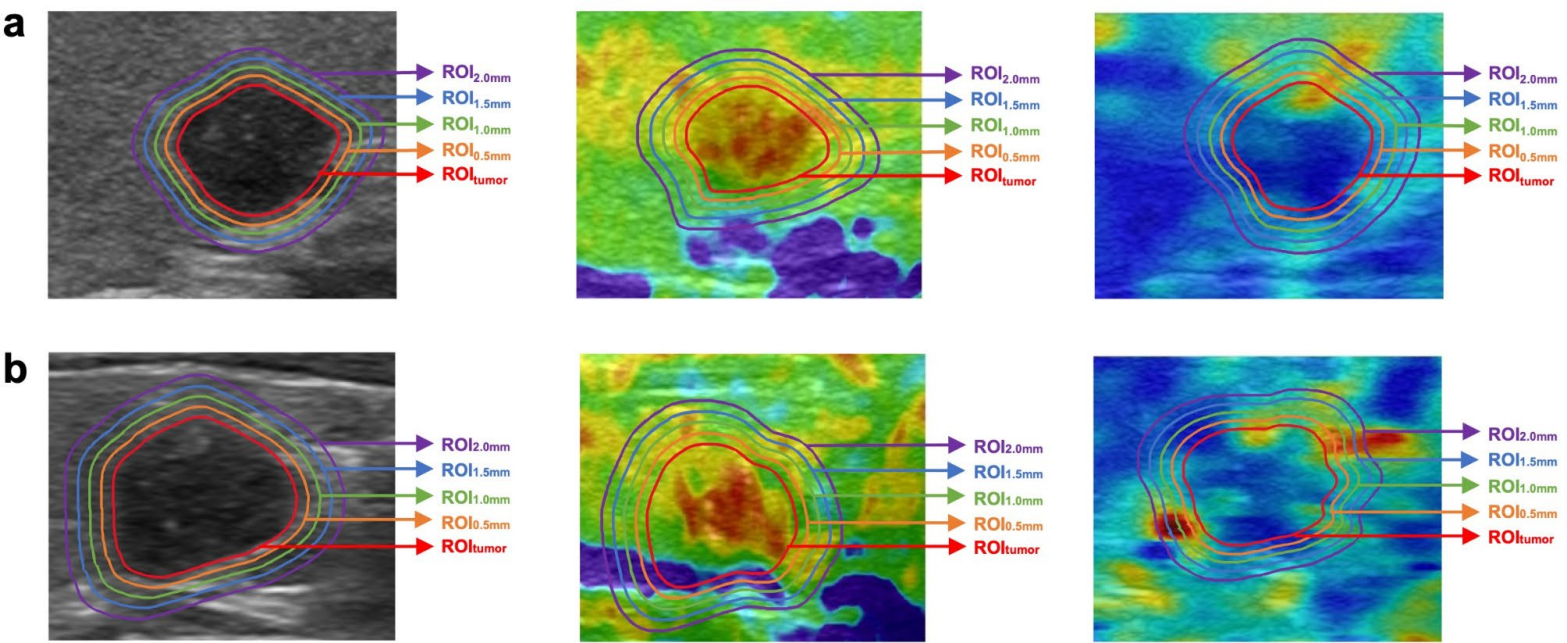
Tumor segmentation and peritumoral expansion of two representative examples on BMUS, SE and SWE images. The ROI_tumor_ represents the intratumoral region. The ROI_0.5 mm_, ROI_1.0 mm_, ROI_1.5 mm_ and ROI_2.0 mm_ represent the combined region including both the intratumoral and peritumoral areas with 0.5–2.0 mm widths. (**a**) A 39-year-old female without cervical LNM; (**b**) A 50-year-old female with cervical LNM. BMUS: B-mode ultrasound; LNM: lymph node metastasis; ROI: region of interest; SE: strain elastography; SWE: shear wave elastography

ROIs were drawn on the tri-modality US images including BMUS, SE, and SWE. First, the ROI_tumor_ representing solely the intratumoral region was manually drawn along the tumor boundary on BMUS by a radiologist with 4 years of experience in thyroid US using ITK-SNAP 3.8.0 (http://www.itksnap.org). The pathological findings and clinical information were not available to the radiologist. Due to the unexplicit margin of the tumor on USE, the ROI_tumor_ on SE and SWE was obtained by mapping the outline of the tumor on BMUS (left screen) to the corresponding USE image (right screen). Then, the dilation codes in Python (version 3.9) were used to automatically extend the peritumoral region. To obtain comprehensive information from the tumor and its surrounding microenvironment, we considered the ROIs as a unified whole for radiomic feature extraction, rather than analyzing intratumoral and peritumoral radiomic features separately. The expansion ROIs of the combined region including both intratumoral and peritumoral areas were denoted as ROI_0.5 mm_, ROI_1.0 mm_, ROI_1.5 mm_ and ROI_2.0 mm_. Radiomics features were extracted from ROIs for each tumor by using open-source platform Pyradiomics (https://pyradiomics.readthedocs.io) according to the Image Biomarker Standardization Initiative (IBSI) [29]. The schematic diagram of ROIs of two representative examples is displayed in Fig. 2.

### Feature selection and radiomics signature construction

**Fig. 3:**
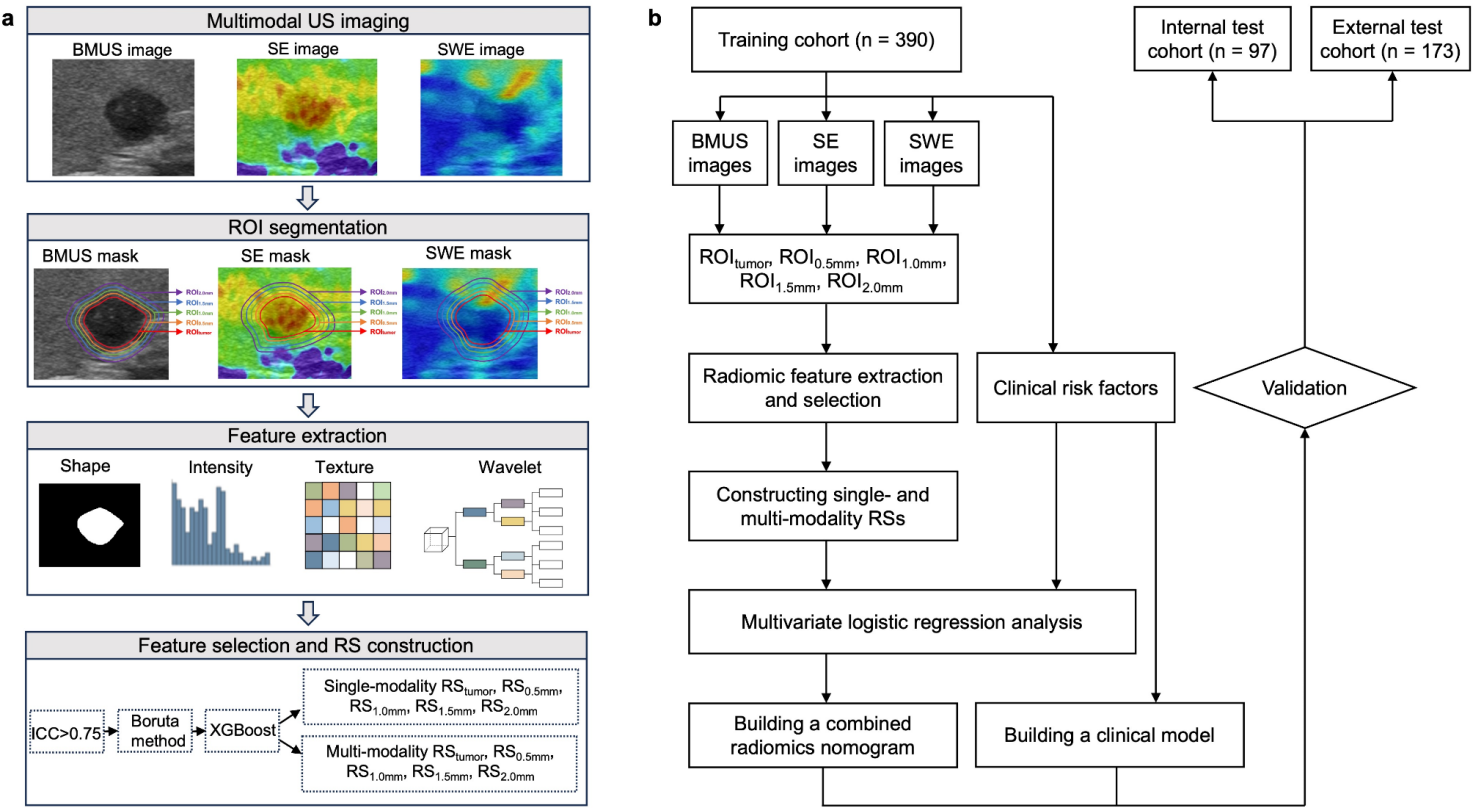
The radiomics workflow (**a**) and overall study design (**b**). BMUS: B-mode ultrasound; ICC: intra-class correlation coefficient; ROI: region of interest; RS: radiomics signature; SE: strain elastography; SWE: shear wave elastography; US: ultrasound

A two-step process was adopted for radiomics feature selection including repeatability analysis and Boruta method. To get rid of the scaling differences, radiomics features were normalized using the z-score normalization method. The intra-class correlation coefficient (ICC) was first used to assess the reproducibility of radiomics feature extraction. Two radiologists re-segmented the randomly-selected 50 patients after two weeks to calculate the intra- and interobserver ICCs. The features with ICCs greater than 0.75 were considered good stability. The Boruta method [30] was then used to calculate each feature’s Shapley value and the max shadow value. When a Shapley value was higher than the max shadow value, the corresponding feature was included in the final feature sets. For each US modality, the selected radiomics features derived from each ROI-subgroup were integrated with the respective corresponding coefficients to build the radiomics signature (RS) by using the XGBoost model, which were denoted as the single-modality RS_tumor_, RS_0.5 mm_, RS_1.0 mm_, RS_1.5 mm_ and RS_2.0 mm_. The multi-modality RSs based on tumor and combined regions named multi-modality RS_tumor_, RS_0.5 mm_, RS_1.0 mm_, RS_1.5 mm_ and RS_2.0 mm_ using the complete feature set were also developed. The area under the receiver operating characteristic curve (AUC) was calculated of each RS, and the final hybrid model was based on the optimal RS with the highest AUC value. The flowchart of the study design is shown in Fig. 3.

### Radiomics nomogram development and evaluation

The univariate logistic regression analysis was used to screen the candidate variables associated with the LNM status in training cohort. The forward stepwise multivariate logistic regression analysis was further adopted to construct a hybrid model on the basis of the optimal RS and the selected corresponding multimodal US characteristics as well as the prominent clinical risk factors. To enhance the practical application, a nomogram was developed based on the hybrid model. Meanwhile, a clinical model using solely clinical independent risk factors were also established.

The performance of the RSs, clinical model and hybrid model was evaluated by AUC, accuracy, sensitivity, specificity, positive predictive value and negative predictive value. The calibration curve and Hosmer-Lemeshow test were used to assess the predictive agreement of the nomogram. The decision curve analysis (DCA) was performed to evaluate the clinical usefulness of the nomogram by quantifying the net benefits at different threshold probabilities. To enhance the interpretability, we used SHapley Additive exPlanations (SHAP) method to assign each feature an importance value for the prediction within the training cohort by calculating average marginal contribution [31]. It’s a visual tool to reveal the relationship between the radiomic features and cervical LNM status.

Two blinded radiologists (a junior radiologist and a senior radiologist with 3 and 9 years of experience in thyroid US diagnosis, respectively) reviewed the datasets in the external test cohort and performed the first round of assessment for cervical LNM. The comparison of the predictive performance of two radiologists to the nomogram in terms of accuracy, sensitivity and specificity within all patients in the external test cohort was conducted. After a one-week wash-out period, two radiologists were asked to reassess the cervical LN status by reviewing the predicted probability provided by the nomogram which was denoted as Nomo score for a second round of assessment. They were given the chance whether to maintain or revise the first assessment. The prediction performance of the radiologists with and without the nomogram’s assistance was also calculated and compared. We also evaluated the predictive ability of the nomogram in the subgroup patients diagnosed as cN0 by the two radiologists and patients with pathologically-diagnosed as central LNM.

### Statistical analysis

All statistical analyses were performed using Python (version 3.9) and IBM SPSS Statistics (version 25). The DeLong’s test was used to compare the AUCs of different models. The McNemar’s *x*^2^ test was used to compare the accuracy, sensitivity and specificity between two radiologists and the hybrid model. The *p*-value < 0.05 represents statistical significance.

## Results

### Baseline characteristics

A total of 660 consecutive PTC patients, including 487 patients from Hospital 1 and 173 from Hospital 2, were finally enrolled in this study. The patients from Hospital 1 between November 2018 and April 2021 were randomly divided into a training cohort (*n* = 390, mean age ± SD, 41 ± 12 years) and an internal test cohort (*n* = 97, mean age ± SD, 41 ± 11 years) at a ratio of 8:2 by the stratified random sampling method. The patients between November 2022 and January 2024 from Hospital 2 were used as the external test cohort (*n* = 173, mean age ± SD, 44 ± 12 years). Pathologically-confirmed cervical LNM occurred in 49.8% (329/660) of all patients. All baseline characteristics are summarized in Table 1. The pathological characteristics of tumors across the three cohorts is shown in Table A.1.

**Table 1:** Baseline characteristics of patients in the training, internal test and external test cohorts

Characteristics	Training cohort (*n* = 390)	Internal test cohort (*n* = 97)	External test cohort (*n* = 173)
Age (y)*	41 ± 11	41 ± 11	43 ± 12
Gender			
Male	86 (22.1)	26 (26.8)	42 (24.3)
Female	304 (77.9)	71 (73.2)	131 (75.7)
Tumor size (mm)*	11.1 ± 6.6	10.4 ± 5.1	10.8 ± 6.0
Primary site			
Left lobe	183 (46.9)	46 (47.4)	82 (47.4)
Right lobe	91 (49.5)	48 (49.5)	80 (46.2)
Isthmus	14 (3.6)	3 (3.1)	11 (6.4)
Shape			
Aspect ratio < 1	249 (63.8)	63 (64.9)	96 (55.5)
Aspect ratio ≥ 1	141 (36.2)	34 (35.1)	77 (44.5)
Microcalcification			
Absent	133 (34.1)	37 (38.1)	71 (41.0)
Present	257 (65.9)	60 (61.9)	102 (59.0)
Composition			
Mixed	30 (7.7)	7 (7.2)	0 (0.0)
Solid	360 (92.3)	90 (92.8)	173 (100.0)
Echogenicity			
Iso or hyperechoic	17 (4.4)	6 (6.2)	10 (5.8)
Hypoechoic	270 (69.2)	72 (74.2)	126 (72.8)
Marked hypoechoic	103 (26.4)	19 (19.6)	37 (21.4)
Margin			
Well-defined	79 (20.3)	18 (18.6)	26 (15.0)
Ill-defined	311 (79.7)	79 (81.4)	147 (85.0)
E_mean_-tumor (kPa)*	29.5 ± 11.2	29.0 ± 11.8	37.5 ± 10.2
E_max_-tumor (kPa)*	75.2 ± 40.0	76.1 ± 40.1	85.3 ± 52.1
E_min_-tumor (kPa)*	9.7 ± 6.5	9.56 ± 7.3	15.3 ± 8.4
E_sd_-tumor (kPa)*	12.2 ± 7.5	11.5 ± 5.9	11.4 ± 6.3
E_mean_-shell_1.0_ (kPa)*	30.8 ± 11.8	21.9 ± 4.5	37.6 ± 11.4
E_max_-shell_1.0_ (kPa)*	79.1 ± 56.9	79.3 ± 43.5	86.9 ± 53.3
E_min_-shell_1.0_ (kPa)*	9.4 ± 6.1	9.9 ± 7.0	14.6 ± 8.8
E_sd_-shell_1.0_ (kPa)*	13.9 ± 8.7	13.3 ± 7.1	13.3 ± 7.3
“Stiff rim” sign			
Absent	127 (32.6)	39 (40.2)	76 (43.9)
Present	263 (67.4)	58 (59.8)	97 (56.1)
B/A*	1.9 ± 0.7	1.8 ± 0.7	2.2 ± 2.5
B/shell_1.0_*	1.3 ± 0.5	1.3 ± 0.5	1.4 ± 0.6
ES			
1–3	120 (30.8)	34 (35.1)	70 (40.5)
4–5	270 (69.2)	63 (64.9)	103 (59.5)

B/A and B/shell_1.0_: strain ratios of normal glandular tissue to the tumor area or the peritumoral area; ES: elastography score

Note: except where indicated, data are numbers of patients, with percentages in parentheses

*Data are means ± SDs.

Table A.2 showed that after the univariate and multivariate logistic regression analyses, age, gender, tumor size, microcalcification, “stiff rim” sign, E_max_-shell_1.0_ and E_sd_-shell_1.0_ remained significant factors for LNM prediction (all *p* < 0.05). Based on these variables, the clinical model yielded the AUCs of 0.750 (95% CI: 0.701, 0.798), 0.777 (95% CI: 0.686, 0.869) and 0.781 (95% CI: 0.708, 0.854) for the training, internal test and external test cohorts, respectively.

### Radiomics feature extraction and selection

The extracted radiomics features could be stratified into the Shape, First Order, Gray-Level Co-occurrence Matrix (GLCM), Gray-Level Size Zone Matrix (GLSZM), Gray-Level Dependence Matrix (GLDM), Gray-Level Run Length Matrix (GLRLM), and Neighborhood Gray-Tone Difference Matrix (NGTDM) features. A number of 944 and 1898 features were contained in each feature set of single and multi-modality US images per patients, respectively. The number of the retained features after each feature selection procedure per ROI from each single modality and multi-modality US images were summarized in Table A.3.

### Radiomics signature performance evaluation and comparison

**Fig. 4:**
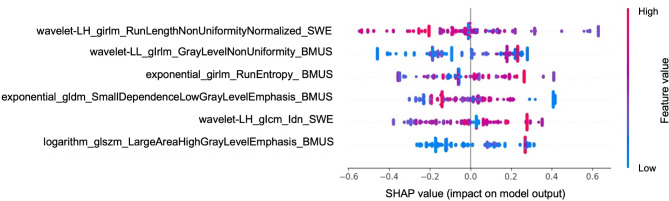
The summary plot of the visual representation of SHAP analysis. It quantified the absolute mean SHAP values which led to the raking of individual contributions of the features within multi-modality RS_1.0 mm_. BMUS: B-mode ultrasound; RS: radiomics signature; SE: strain elastography; SHAP: SHapley Additive exPlanations; SWE: shear wave elastography

Table 2 listed the performance of multi-modality RSs with different widths across the three cohorts and the receiver operating characteristic (ROC) curves are depicted in Figure A.1. The results indicated that the multi-modality RS_1.0 mm_ yielded the most predictive performance with the AUCs of 0.862 (95% CI: 0.825, 0.899), 0.798 (95% CI: 0.708, 0.887) and 0.789 (95% CI: 0.720, 0.857) in the training, internal test and external test cohorts, respectively. The detailed selected features and the corresponding coefficients of multi-modality RS_1.0 mm_ were presented in Table A.4. The SHAP was depicted as Fig. 4 to enhance the explainability. DeLong’s test showed that within the multi-modal subgroups, the performance of RS_1.0 mm_ were significantly higher than that of RS_0.5 mm_, RS_1.5 mm_ and RS_2.0 mm_ in training (AUCs: 0.862 vs. 0.777; 0.862 vs. 0.712 and 0.862 vs. 0.714; all *p* < 0.001) and external test cohorts (AUCs: 0.789 vs. 0.644, *p* < 0.001; 0.789 vs. 0.673; *p* = 0.006 and 0.789 vs. 0.688; *p* = 0.009). The multi-modality RS_1.0 mm_ also had higher AUCs than those of other RSs in the internal test cohort (AUCs: 0.798 vs. 0.760, *p* = 0.44; 0.798 vs. 0.734, *p* = 0.17; and 0.798 vs. 0.662, *p* = 0.02).

**Table 2:** Performance of the multi-modality radiomics signatures derived from the intratumoral and combined regions with different widths in the training, internal test and external test cohorts

Cohort	Tumor region	AUC (95% CI)	ACC (%)	SEN (%)	SPE (%)	PPV (%)	NPV (%)
Training cohort	ROI_tumor_	0.678 (0.625, 0.730)	63.3 (247/390)	73.9 (153/207)	51.4 (94/183)	63.2 (153/242)	63.5 (94/148)
	ROI_0.5 mm_	0.777 (0.731, 0.822)	71.3 (278/390)	73.4 (152/207)	68.9 (126/183)	72.7 (152/209)	69.6 (126/181)
	ROI_1.0 mm_	0.862 (0.825, 0.899)	80.0 (312/390)	81.2 (168/207)	78.7 (144/183)	81.2 (168/207)	78.7 (144/183)
	ROI_1.5 mm_	0.712 (0.660, 0.763)	66.7 (260/390)	71.0 (147/207)	61.8 (113/183)	67.7 (147/217)	65.3 (113/173)
	ROI_2.0 mm_	0.714 (0.663, 0.764)	64.4 (251/390)	58.5 (121/207)	71.0 (130/183)	69.5 (121/174)	60.2 (130/216)
Internal test cohort	ROI_tumor_	0.682 (0.575, 0.789)	67.0 (65/97)	74.5 (38/51)	58.7 (27/46)	66.7 (38/57)	67.5 (27/40)
	ROI_0.5 mm_	0.760 (0.665, 0.855)	69.1 (67/97)	70.6 (36/51)	67.4 (31/46)	70.6 (36/51)	67.4 (31/46)
	ROI_1.0 mm_	0.798 (0.708, 0.887)	76.3 (74/97)	68.6 (35/51)	84.8 (39/46)	83.3 (35/42)	70.9 (39/55)
	ROI_1.5 mm_	0.734 (0.635, 0.832)	63.9 (62/97)	66.7 (34/51)	60.9 (28/46)	65.4 (34/52)	62.2 (28/45)
	ROI_2.0 mm_	0.662 (0.554, 0.771)	63.9 (62/97)	52.9 (27/51)	76.1 (35/46)	71.1 (27/38)	59.3 (35/59)
External test cohort	ROI_tumor_	0.665 (0.583, 0.747)	63.6 (110/173)	33.8 (24/71)	84.3 (86/102)	60.0 (24/40)	64.7 (86/133)
	ROI_0.5 mm_	0.644 (0.560, 0.724)	61.9 (107/173)	36.6 (26/71)	79.4 (81/102)	55.3 (26/47)	64.3 (81/126)
	ROI_1.0 mm_	0.789 (0.720, 0.857)	72.8 (126/173)	54.9 (39/71)	85.3 (87/102)	72.2 (39/54)	73.1 (87/119)
	ROI_1.5 mm_	0.673 (0.588, 0.757)	68.2 (118/173)	49.3 (35/71)	81.4 (83/102)	64.8 (35/54)	69.7 (83/119)
	ROI_2.0 mm_	0.688 (0.606, 0.771)	65.9 (114/173)	47.9 (34/71)	78.4 (80/102)	60.7 (34/56)	68.4 (80/117)

ACC: accuracy; AUC: area under the ROC curve; CI: confidence interval; NPV: negative predictive value; PPV: positive predictive value; ROI: region of interest; SEN: sensitivity; SPE: specificity

As shown in Table 2, within a 1.0 mm range, the RS_1.0 mm_ combining intratumoral and peritumoral region significantly outperformed the RS_tumor_ for the intratumoral region alone in the training (0.862 vs. 0.678, *p* < 0.001), internal test (0.798 vs. 0.682, *p* = 0.036), and external test cohort (0.789 vs. 0.665, *p* = 0.002), respectively. In other ranges, most of the RSs based on combined regions also outperformed RS_tumor_ alone across the three cohorts, with a few differences reaching statistical significance.

Table 3 listed the performance of single-modality RS_1.0 mm_ and multi-modality RS_1.0 mm_ across the three cohorts and the ROC curves are depicted in Figure A.2. It showed that the performance of the multimodal RSs was significantly superior to the RSs based on each individual modality in the training (0.862 vs. 0.694 for BMUS; 0.862 vs. 0.629 for SE; 0.862 vs. 0.822 for SWE, all *p* < 0.05) and external test cohorts (0.789 vs. 0.688 for BMUS; 0.789 vs. 0.599 for SE; 0.789 vs. 0.642 for SWE, all *p* < 0.05). This was also observed in the internal test cohort (0.798 vs. 0.705 for BMUS, *p* = 0.10; 0.798 vs. 0.697 for SE, *p* = 0.09; 0.798 vs. 0.725 for SWE, *p* = 0.09).

**Table 3:** Performance of single-modality and multi-modality radiomics signatures based on ROI_1.0 mm_ in the training, internal test and external test cohorts

Cohort	Modality	AUC (95% CI)	ACC (%)	SEN (%)	SPE (%)	PPV (%)	NPV (%)
Training cohort	BMUS	0.694 (0.642, 0.746)	66.2 (258/390)	72.0 (149/207)	59.6 (109/183)	66.8 (149/223)	65.3 (109/167)
	SE	0.629 (0.574, 0.685)	62.1 (242/390)	65.2 (135/207)	58.5 (107/183)	64.0 (135/211)	59.8 (107/179)
	SWE	0.822 (0.781, 0.863)	73.8 (288/390)	74.9 (155/207)	72.7 (133/183)	75.6 (155/205)	71.9 (133/185)
	BMUS + SE + SWE	0.862 (0.825, 0.899)	80.0 (312/390)	81.2 (168/207)	78.7 (144/183)	81.2 (168/207)	78.7 (144/183)
Internal test cohort	BMUS	0.705 (0.600, 0.810)	69.1 (67/97)	68.6 (35/51)	69.6 (32/46)	71.4 (35/49)	66.7 (32/48)
	SE	0.697 (0.589. 0.805)	66.0 (64/97)	64.7 (33/51)	67.4 (31/46)	68.8 (33/48)	63.3 (31/49)
	SWE	0.725 (0.625, 0.825)	65.0 (63/97)	66.7 (34/51)	63.0 (29/46)	66.7 (34/51)	63.0 (29/46)
	BMUS + SE + SWE	0.798 (0.708, 0.887)	76.3 (74/97)	68.6 (35/51)	84.8 (39/46)	83.3 (35/42)	70.9 (39/55)
External test cohort	BMUS	0.688 (0.610, 0.767)	63.0 (109/173)	47.9 (34/71)	73.5 (75/102)	55.7 (34/61)	67.0 (75/112)
	SE	0.599 (0.512, 0.686)	61.9 (108/173)	35.2 (25/71)	80.4 (82/102)	55.6 (25/45)	64.1 (82/128)
	SWE	0.642 (0.557, 0.728)	60.7 (105/173)	33.8 (24/71)	79.4 (81/102)	53.3 (24/45)	63.3 (81/128)
	BMUS + SE + SWE	0.789 (0.720, 0.857)	72.8 (126/173)	54.9 (39/71)	85.3 (87/102)	72.2 (39/54)	73.1 (87/119)

ACC: accuracy; AUC: area under the ROC curve; BMUS: B-mode ultrasound; CI: confidence interval; SE: strain elastography; SEN: sensitivity; SPE: specificity; SWE: shear wave elastography

### Radiomics nomogram performance evaluation and comparison

**Fig. 5:**
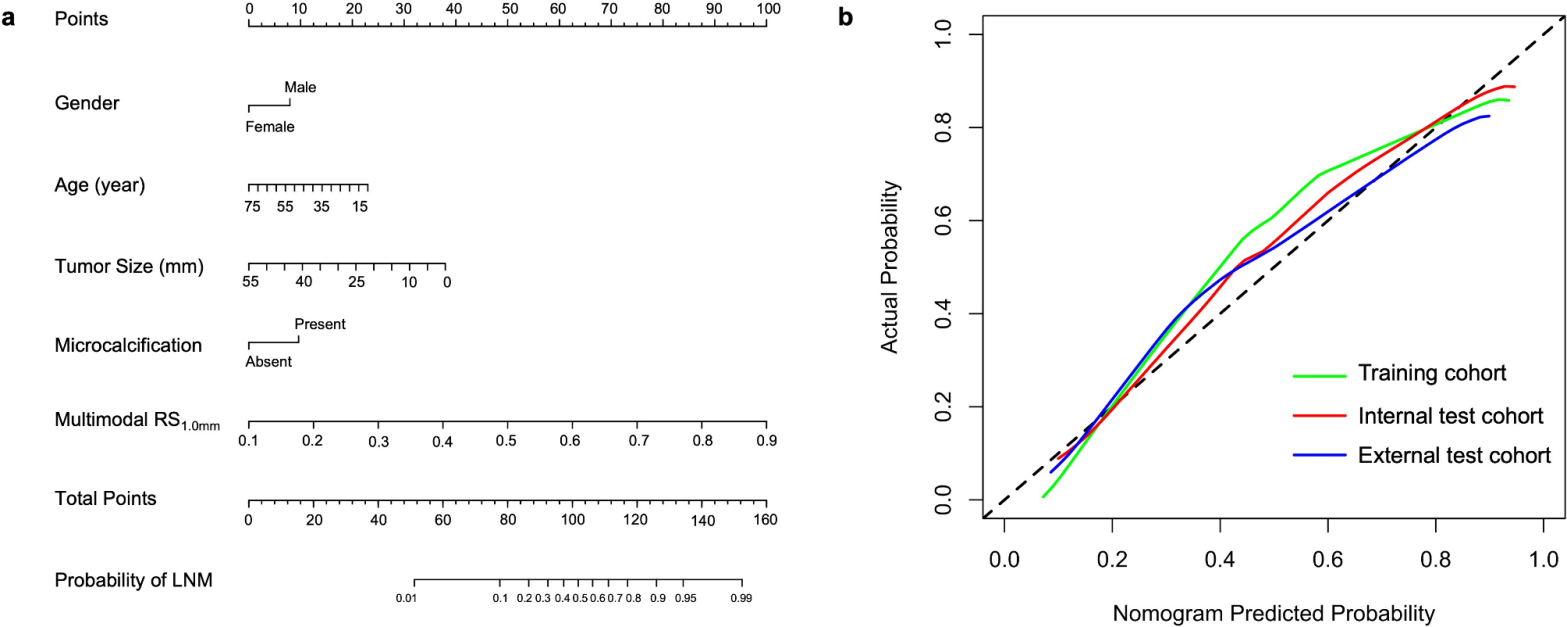
The nomogram and calibration curves of the training, internal test, and external test cohorts. (**a**) Nomogram for predicting the probability of cervical LNM in PTC patients; (**b**) Calibration curves of the nomogram across the three cohorts. LNM: lymph node metastasis; PTC: papillary thyroid carcinoma; RS_1.0 mm_: radiomics signature based on intratumoral and 1 mm peritumoral region

As the RS_1.0 mm_ derived from the multimodal imaging achieved the highest predictive performance, it was incorporated to the final hybrid model as the optimal RS. The forward stepwise multivariate logistic regression analysis confirmed age, gender, tumor size, microcalcification and multi-modality RS_1.0 mm_ as the independent risk predictors of the hybrid model for cervical LNM prediction in PTC. A nomogram was then depicted based on the hybrid model as presented in Fig. 5a. The calibration curve was displayed in Fig. 5b, and the Hosmer-Lemeshow test indicated no significant deviations between the observed and predicted curves both in the internal (*p* = 0.151) and external test cohorts (*p* = 0.718). The optimal cut-off value of the risk probabilities which was denoted as Nomo-score by the nomogram was 0.391 via maximizing the Youden index.

**Fig. 6:**
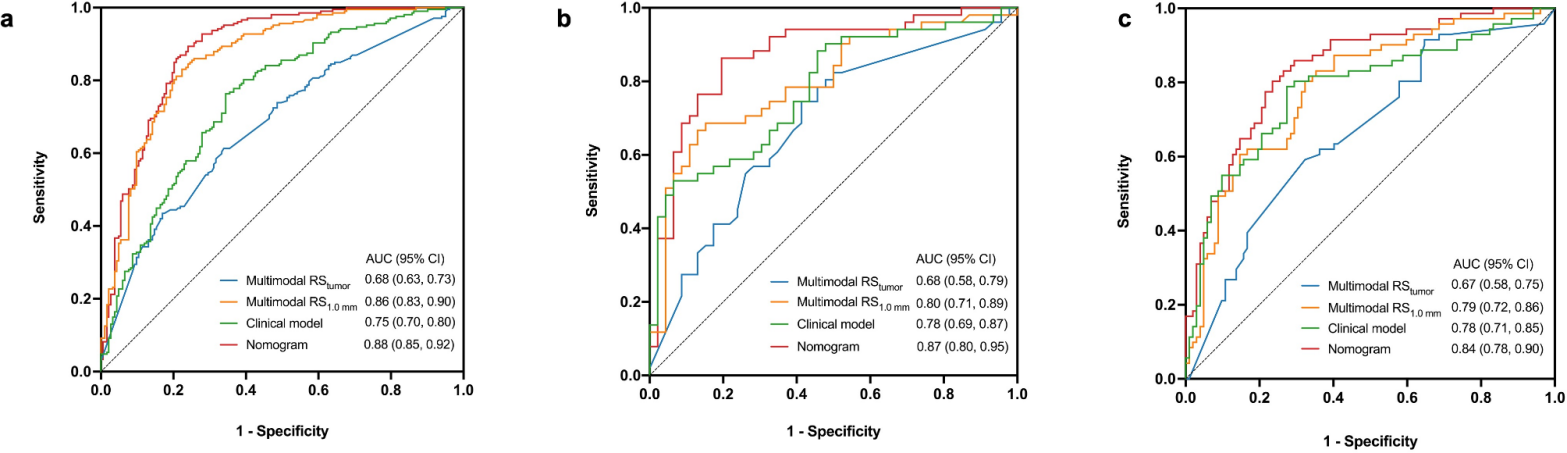
Receiver operating characteristic (ROC) curves of different models for predicting cervical LNM in PTC for the (**a**) training cohort, (**b**) internal test cohort and (**c**) external test cohort

As shown in Table 4; Fig. 6, the hybrid model exhibited AUCs of 0.883 (95% CI: 0.849, 0.918), 0.873 (95% CI: 0.799, 0.946) and 0.841 (95% CI: 0.782, 0.900) for the training, internal test and external test cohorts, respectively. The hybrid model demonstrated superior predictive efficiency than the clinical model, multimodal RS_tumor_ and RS_1.0 mm_ for the training cohort (AUCs: 0.883 vs. 0.750, 0.678, 0.862, respectively; all *p* < 0.05), internal test cohort (AUCs: 0.873 vs. 0.777, 0.682, 0.798, respectively; all *p* < 0.05) and external test cohort (AUCs: 0.841 vs. 0.781, 0.665, 0.789, respectively; all *p* < 0.05). The DCA indicated that the nomogram’s overall net benefit in predicting LNM was higher than that of the clinical model and the multimodal RSs across most reasonable threshold probabilities (Figure A.3). Moreover, within the internal and external test cohorts, the patients were divided into the high-risk (Nomo score > 0.391) and low-risk (Nomo score < 0.391) subgroups. The high-risk subgroup had a significantly higher proportion of LNM-positive patients across both cohorts (all *p* < 0.001) (Figure A.4).

**Table 4:** Performance of multi-modality RS_tumor_, multi-modality RS_1.0 mm_, clinical model and nomogram in the training, internal test and external test cohorts

Cohort	Model	AUC (95% CI)	ACC (%)	SEN (%)	SPE (%)	PPV (%)	NPV (%)
Training cohort	Multimodality RS_tumor_	0.678 (0.625, 0.730)	63.3 (247/390)	73.9 (153/207)	51.4 (94/183)	63.2 (153/242)	63.5 (94/148)
	Multimodality RS_1.0 mm_	0.862 (0.825, 0.899)	80.0 (312/390)	81.2 (168/207)	78.7 (144/183)	81.2 (168/207)	78.7 (144/183)
	Clinical model	0.750 (0.701, 0.798)	70.5 (275/390)	74.9 (155/207)	65.6 (120/183)	71.1 (155/218)	69.8 (120/172)
	Nomogram	0.883 (0.849, 0.918)	80.8 (315/390)	81.2 (168/207)	80.3 (147/183)	82.4 (168/204)	79.0 (147/186)
Internal test cohort	Multimodality RS_tumor_	0.682 (0.575, 0.789)	67.0 (65/97)	74.5 (38/51)	58.7 (27/46)	66.7 (38/57)	67.5 (27/40)
	Multimodality RS_1.0 mm_	0.798 (0.708, 0.887)	76.3 (74/97)	68.6 (35/51)	84.8 (39/46)	83.3 (35/42)	70.9 (39/55)
	Clinical model	0.777 (0.686, 0.869)	66.0 (64/97)	68.6 (35/51)	63.0 (29/46)	67.3 (35/52)	64.4 (29/45)
	Nomogram	0.873 (0.799, 0.946)	83.5 (81/97)	86.3 (44/51)	80.4 (37/46)	83.0 (44/53)	84.1 (37/44)
External test cohort	Multimodality RS_tumor_	0.665 (0.583, 0.747)	63.6 (110/173)	33.8 (24/71)	84.3 (86/102)	60.0 (24/40)	64.7 (86/133)
	Multimodality RS_1.0 mm_	0.789 (0.720, 0.857)	72.8 (126/173)	54.9 (39/71)	85.3 (87/102)	72.2 (39/54)	73.1 (87/119)
	Clinical model	0.781 (0.708, 0.854)	73.4 (127/173)	64.8 (46/71)	79.4 (81/102)	68.7 (46/67)	76.4 (81/106)
	Nomogram	0.841 (0.782, 0.900)	77.5 (134/173)	78.9 (56/71)	76.5 (78/102)	70.0 (56/80)	83.9 (78/93)

ACC: accuracy; AUC: area under the ROC curve; RS: radiomics signature; SEN: sensitive; SPE: specificity

As shown in Table 5, in the external test cohort, the predictive accuracy of nomogram was significantly higher than that of the junior radiologist (77.5% vs. 58.4%, *p* < 0.001) and the senior radiologist (77.5% vs. 65.9%, *p* = 0.032). The nomogram generated significantly higher sensitivity than that of the junior (78.9% vs. 50.7%, *p* = 0.001) and the senior radiologist (78.9% vs. 59.2%, *p* = 0.022). The nomogram-assisted strategy significantly increased the accuracies of the junior and senior radiologists from 58.4 to 69.4% (*p* < 0.001) and from 65.9 to 74.0% (*p* = 0.001), respectively. The nomogram’s assistance also significantly improved the sensitivities of junior radiologists from 50.7 to 70.4% (*p* = 0.001) and senior radiologists from 59.2 to 77.5% (*p* < 0.001).

**Table 5:** The performance of nomogram, radiologists without and with nomogram assistance in the external test cohort

	Nomogram	R1	*p*-value (R1 vs. nomogram)	R1 with nomogram	*p*-value (R1 vs. R1 with nomogram)	R2	*p*-value (R2 vs. nomogram)	R2 with nomogram	*p*-value (R2 vs. R2 with nomogram)
ACC (%)	77.5 (134/173)	58.4 (101/173)	< 0.001	69.4 (120/173)	< 0.001	65.9 (114/173)	0.032	74.0 (128/173)	0.001
SENS (%)	78.9 (56/71)	50.7 (36/71)	0.001	70.4 (50/71)	0.001	59.2 (42/71)	0.022	77.5 (55/71)	< 0.001
SPEC (%)	76.5 (78/102)	63.7 (65/102)	0.049	68.6 (70/102)	0.074	70.6 (72/102)	0.461	72.5 (74/102)	0.480

Abbreviations: ACC, accuracy; R1, junior radiologist; R2, senior radiologist; SENS, sensitivity; SPEC specificity

Moreover, the nomogram yielded AUCs of 0.859 (95% CI: 0.779, 0.940) and 0.840 (95% CI: 0.759, 0.920) for the cN0 subgroup diagnosed by junior radiologist (*n* = 100) and senior radiologist (*n* = 101), respectively. The ROCs of the nomogram in the cN0 subgroups are shown in Figure A.5. Within the central LNM subgroup, the sensitivity of nomogram was significantly higher than that of junior (79.4% vs. 48.5%, *p* < 0.001) and senior radiologist (79.4% vs. 57.4%, *p* = 0.012). Under the assistance of the nomogram, the sensitivities of junior and senior radiologists improved from 48.5 to 69.1% (*p* < 0.001) and from 57.4 to 76.5% (*p* < 0.001).

## Discussion

In this bi-central study, our findings indicated that RS_1.0 mm_ derived from multimodal US imaging combining intratumoral and 1 mm peritumoral regions significantly outperformed RSs based on other combined and intratumoral regions for predicting cervical LNM in PTC. Hence, a hybrid model integrating multimodal RS_1.0 mm_ and US-clinical risk predictors was developed and a nomogram was crafted. Notwithstanding the relatively acceptable performance of the clinical model, the hybrid model presented the strongest predictive power for cervical LNM prediction with AUCs of 0.841 to 0.883 across the three cohorts. The hybrid model, presented as a nomogram, can assist to improve the predictive accuracy and sensitivity for radiologists.

Accurate assessment of cervical LNM for PTC can facilitate the informed management decisions in clinical settings [5]. As a non-invasive biomarker by extracting numerous quantitative features, radiomics method has been extensively applied to predict cervical LNM in PTC [32–35]. However, these studies predominantly concentrated on the tumor region, thereby disregarding the diagnostic value of the peritumoral area that plays a pivotal role in tumor microenvironment [36]. Tumor microenvironment encompasses the immediate cellular area surrounding the tumor, which includes blood vessels, immune cells, fibroblasts, signaling molecules, and extracellular matrix [37]. Tumor cells are intimately connected to the adjacent immune cells, engaging in constant interactions. The significant biological interactions occur in the peritumoral area, which are critical for tumor initiation, progression and metastasis [38]. Recently, a growing body of researches has suggested that the peritumoral area harbors valuable information in diagnosing thyroid nodules [39] and breast masses [25], predicting LNM in breast cancer and cervical cancer [40, 41], predicting microvascular invasion status [42] and treatment responsiveness [43, 44]. In our study, the majority of the performance of RS_0.5 mm_, RS_1.0 mm_, RS_1.5 mm_ and RS_2.0 mm_ derived from combined regions surpassed that of the RS_tumor_ across the three cohorts. The result was in tandem with the study of Zhang et al. [45], which has shown the enhanced performance of combined intratumoral and peritumoral radiomics models for axillary LNM prediction in breast cancer. This can be explained by that the higher density of lymphatic vessels in peritumoral areas may contribute to elevated regional LNM [46]. Previous study has shown that a rise in lymphatic vessel density can alter the tumor microenvironment and enhance metastatic potential [47]. These changes are critical for understanding metastatic behavior which cannot be captured by intratumoral features alone. Hence, peritumoral features may serve as indicators of the tumor’s invasiveness. The integration of intra- and peritumoral features offers a more comprehensive characterization of the tumor’s biology, leading to more accurate prediction.

Previously, only one study has evaluated the potential of peritumoral radiomic features for LNM prediction in PTC using contrast-enhanced CT [48]. However, this study was limited to a single-center setting with a small sample size. Besides, the peritumoral expansion size was fixed as 3–5 mm, which was considered too broad for thyroid tumors. Ding et al. suggested to systematically compare different peritumoral widths to identify the optimal size since the selection of peritumoral size might influence the prediction outcomes of the radiomics pipeline [49]. In our bi-center study, we found that multi-modality RS_1.0 mm_ based on the 1 mm peritumoral size could maximize the assessment of LNM status with AUCs of 0.862, 0.798 and 0.789 in the training, internal test and external test cohorts, outperforming the RS subgroups based on other peritumoral sizes. Additionally, our findings indicated that in both internal and external test cohorts, the efficacy of RS_0.5 mm_ and RS_2.0 mm_ was compromised when the expansion range was either excessively large or minimal, which was even inferior to the RS_tumor_. This was probably due to that the large or minimal expansion sizes could dilute the relevant features with too much surrounding normal tissue or lose detail by focusing too narrowly on the tumor. The superior performance of the RS_1.0 mm_ compared to larger or smaller ROIs suggested that this specific range struck a balance between capturing sufficient contextual information to enhance predictive features and minimizing the inclusion of unrelated tissue that could introduce noise. This finding further highlighted the need for precise ROI selection in radiomics studies to ensure the capture of meaningful and relevant features. By systematically evaluating different peritumoral margins, our study provides a methodological framework that can be applied to other cancers where the tumor microenvironment plays a critical role in metastasis. This approach can uncover novel radiomics biomarkers and deepen our understanding of tumor biology, ultimately contributing to more effective diagnostic and therapeutic strategies.

Although multimodal radiomics has been explored in PTC, the combined use of BMUS and USE for intra- and peritumoral feature extraction has not yet been investigated. The USE can provide stiffness measurements that reflect tissue biomechanical properties, which are closely associated with malignancy and metastatic potential. One past study explored the radiomics features derived from the BMUS and SWE images of primary tumors for predicting LNM in PTC [35]. However, the AUC of the nomogram using RS based on SWE features and other risk clinical features was limited as 0.832 in the validation cohort. In the present study, the developed hybrid model in our study generated AUCs of 0.873 and 0.841 in the internal test and external test cohorts. Furthermore, the integration of multimodal radiomics features significantly outperformed features derived from any single modality within ROI_1.0 mm_. The improvements can be attributed to the complementary information and diversity in features provided by each imaging modality, emphasizing their collective utility in improving LNM prediction accuracy. Combining these modalities allows for a more holistic and robust characterization of the tumor, which captures diverse aspects of the tumor’s biological behavior. However, while we initially considered SE features for multimodal radiomics analysis, these features were excluded from final multimodal RS_1.0 mm_. This may be due to that SE features might have exhibited redundancy with those from BMUS and SWE during the feature selection process since SE features provides similar information regarding tumor stiffness to SWE. Moreover, the reliance on manual compression of SE could introduce variability, leading the less predictive power and consistency. The hand pressure could potentially lead to an elevation of stiffness values, particularly in superficial thyroid tumors, which may potentially affect the reliability of the results.

Moreover, we established a clinical model based on the significant predictors of age, gender, tumor size, microcalcification, “stiff rim” sign, E_max_-shell_1.0_ and E_sd_-shell_1.0_ for LNM prediction in PTC. The clinical model had acceptable AUCs of 0.750, 0.777 and 0.781 across the three cohorts. Recent researches have associated peritumoral stiffness with biological behavior of tumor [17, 18, 50]. However, no study has yet explored the predictive potential of peritumoral stiffness in forecasting cervical LNM status in PTC. To the best of our knowledge, our study was the first to find that maximum stiffness value and SD of stiffness within the peritumoral 1 mm region, namely E_max_-shell_1.0_ and E_sd_-shell_1.0_, served as independent risk factors for predicting LNM in PTC. The studies of Zhao et al. [17] and Zhang et al. [51] have also substantiated the prognostic utility of the maximum stiffness value in the peritumoral region assessed by USE. This can be explained by that cancer cells tend to infiltrate into the surrounding tissues as the disease progresses, resulting in the higher stiffness of peritumoral tissues [15]. Elevated stiffness values often correlate with increased fibrosis, desmoplastic reaction, and possibly a higher density of malignant cells. These factors indicate more aggressive tumor behavior and a higher likelihood of metastasis. Furthermore, we found that “stiff rim” sign was significantly correlated with the cervical LNM of PTC, which is in tandem with Zhang et al. [51].

Despite of the acceptable performance of clinical model, a hybrid model integrating multimodality RS_1.0 mm_ with clinical parameters exhibited excellent predictive ability and calibration with AUCs of 0.883, 0.873 and 0.841 across the three cohorts, which significantly outperformed the clinical model, multimodality RS_tumor_ and RS_1.0 mm_. The DCA further revealed that using the nomogram to predict cervical LNM status offered superior overall net benefits compared to the clinical model, RSs, and the “treat all” or “treat none” strategies across most threshold probabilities. This suggested a synergistic effect where clinical variables provided a broad overview based on well-established risk factors of essential patient-specific information, while radiomics features offered detailed insights into the nuanced and quantitative aspects of tumor imaging that were not apparent in the routine clinical assessments. Moreover, we divided the patients into high-risk and low-risk subgroups according to the optimal cut-off value of the risk probabilities of the hybrid model. The high-risk subgroup had a significantly higher proportion of LNM-positive patients, further facilitating the personalized treatment planning. Hence, by leveraging the nomogram based on the hybrid model, patients could be more accurately categorized into risk groups, enabling tailored therapeutic approaches that improved outcomes and reduced unnecessary interventions.

According to the 2015 ATA guideline, therapeutic LND is recommended for patients with apparent LNM that are either cytologically confirmed or highly suspicious. However, preoperative evaluation of cervical LNM status by radiologists is not only limited in sensitivity but also highly dependent on the experience of the radiologists. A major advantage of the nomogram is its ability to significantly enhance the sensitivity of preoperative LNM evaluation. In the external test cohort, the nomogram’s sensitivity was higher than that of both junior and senior radiologists. By referring to the Nomo score provided by the nomogram, the accuracies and sensitivities were significantly improved for both junior and senior radiologists. This is particularly important in the clinical management of PTC, where early detection of LNM is critical for determining the most appropriate surgical approach. By increasing sensitivity, the nomogram may help reduce the risk of missed diagnoses. The ability of the nomogram to consistently provide accurate predictions supports its utility as a reliable decision-support tool for clinicians managing PTC patients. For patients with PTC who are diagnosed with high risk of LNM by the nomogram through preoperative multi-modality imaging, it can help determine whether more extensive surgery, such as total thyroidectomy and LND, is warranted. Accordingly, low-risk patients can benefit from non-surgical management, helping to avoid overtreatment and to reduce unnecessary surgeries or postoperative complications.

The performance of the nomogram was also evaluated in subgroups with more specific clinical challenges where LNM is difficult to detect. In our study, 35.0% (35/100) and 28.7% (29/101) of the patients who were initially diagnosed as cN0 by junior and senior radiologists were later found to have LNM upon pathological examination. In the cN0 subgroup, the nomogram exhibited robust performance with AUCs of 0.859 and 0.840 for junior and senior radiologists, respectively. These AUCs suggest that the nomogram is effective in stratifying risk even when clinical examination and US imaging fail to detect LNM. In the central LNM subgroup, where US detection can be particularly challenging due to the anatomical limitations of the central neck region, the nomogram’s sensitivity was significantly higher than that of the junior and senior radiologists. These findings demonstrate that the nomogram can improve the diagnosis of LNM in the central neck, which is often undetected in clinical practice due to its subtle presentation on imaging.

There are certain limitations in the study. First, it is essential to validate these findings in larger prospective cohorts to ensure the generalizability. Second, exploring the biological underpinnings of the observed differences in predictive performance across various ROIs can provide deeper insights into tumor biology and its interaction with surrounding tissues. Moreover, due to the need for a sufficient tumor surrounding area for analysis, the study lacked larger tumors. This may also explain why tumor size had relatively little significance in the current nomogram for the predictive model. The ROIs were manually delineated by radiologists, which was labor-intensive and time-consuming, leading to potential inter-observer variability. Future study can leverage deep learning techniques for automated segmentation and feature extraction, which will streamline the radiomic workflow and improve accuracy. Finally, to address the variability of surgeries among different hospitals, the standardized surgical procedures including the selection of thyroidectomy and cervical LND in the two participating hospitals strictly followed the 2015 ATA guideline [5]. However, the variability in LND practices across different hospitals which may have influenced the detection rate of cervical LNM could not be fully controlled due to variations in physician experience and patient preferences, etc.

## Conclusions

In conclusion, the study provided robust evidence supporting the efficacy of the combination of intra- and peritumoral radiomic features derived from multimodality US imaging, particularly within a 1 mm margin, to predict cervical LNM in PTC. The peritumoral elasticity measurements were demonstrated to be the independent predictors of LNM. Combining radiomic data with clinical features in a prediction model further improves the prediction of cervical LNM, which enables more precise and individualized treatment strategies for PTC patients. The nomogram complements clinicians by providing additional predictive insights. These findings not only provide a foundation for future research exploring the tumor microenvironment’s role in cancer progression but also enhance the diagnostic utility of multimodal US imaging.

## Electronic supplementary material

Below is the link to the electronic supplementary material.

Supplementary Material 1

## Data Availability

The datasets used and analyzed during the current study are available from the corresponding author on reasonable request.

## Abbreviations

ACR TIRADS: American College of Radiology Thyroid Imaging Reporting and Data System
ATA: American Thyroid Association
AUC: Area under the receiver operating characteristic curve
BMUS: B-mode ultrasound
cN0: Clinically node-negative
DCA: Decision curve analysis
ES: Elasticity score
GLCM: Gray-Level Co-occurrence Matrix
GLDM: Gray-Level Dependence Matrix
GLRLM: Gray-Level Run Length Matrix
GLSZM: Gray-Level Size Zone Matrix
ICC: Intra-class correlation coefficient
LN: Lymph node
LND: Lymph node dissection
LNM: Lymph node metastasis
NGTDM: Neighborhood Gray-Tone Difference Matrix
PTC: Papillary thyroid carcinoma
ROC: Receiver operating characteristic
ROI: Region of interest
RS: Radiomics signature
SD: Standard deviation
SE: Strain elastography
SHAP: SHapley Additive exPlanations
SWE: Shear wave elastography
SR: Strain ratio
XGBoost: Extreme gradient boosting
US: Ultrasound
USE: Ultrasound elastography
