# Supplementary material for: Predicting lymph node metastasis in papillary thyroid carcinoma: radiomics using two types of ultrasound elastography
Source: Cancer Imaging. 2025 Feb 13;25:13. doi: 10.1186/s40644-025-00832-w (PMC11827213; doi:10.1186/s40644-025-00832-w)
Supplement: Supplementary file 1 — Supplementary Material 1 [file 40644_2025_832_MOESM1_ESM.docx]

**Appendix A.1 Multimodality ultrasound imaging protocol**

The BMUS, SE and SWE examinations of each target thyroid tumor were performed using Resona 7 (Mindray Medical International, Shenzhen, China) with a line array probe (L14-5 W) at a frequency of 4.0-14.0 MHz by a board-certified radiologist with 10 years of experience in thyroid US and 5 years of experience in USE within Hospital 1. Similarly, Resona 7 and Resona DC-8 US systems (Mindray Medical International, Shenzhen, China) was used by a board-certified radiologist with 8 years of experience in thyroid US and 5 years of experience in USE for tri-modality US imaging within Hospital 2. Patients were kept in a supine position with a fully exposed neck during the examination. Conventional US examination was first performed. Subsequent SE and SWE examinations were performed by the same radiologist at the same plane with the patient’s position remaining unchanged. The images of the target tumor were stored into the Picture Archiving and Communication Systems (PACS) for subsequent analysis.

Two radiologists with over 10 years of experience in thyroid US independently conducted blinded reviews of all images. Any discrepancies were resolved by a consensus decision. The BMUS characteristics were analyzed and the American College of Radiology Thyroid Imaging Reporting and Data System (ACR TIRADS) category was assigned when images were acquired. The BMUS characteristics of target tumor included size, primary site, composition, echogenicity, margin, shape and microcalcification.

For SE, a rectangular region of interest (ROI) box was focused on the target tumor and adjusted to include the subcutaneous fat layer to the superficial portion of the pectoralis muscle layer. The subtle manual compression was vertically compressed by the transducer on target tumor. The patients were instructed to hold their breath. The satisfactory SE image was acquired and saved when the green control bar at the bottom of the screen was stable. The elasticity score (ES), a qualitative elasticity parament, was determined using the five-point scoring system [1]: 1 = even strain over the entire lesion; 2 = strain in most of the lesion; 3 = strain at the periphery of the lesion with sparing in the center of a lesion; 4 = no strain in the entire lesion; 5 = no strain in the entire lesion or the surrounding area. The strain ratio (SR), a semi-quantitative SE index, which calculated the average strain of target tumor or peritumoral tissue and compared it to the average strain of the adjacent normal glandular tissue at a similar depth to the target tumor, was also measured using the embedded software program in the US system. Three regions of interests (ROIs) were acquired: ROI (A) was outlined along the border of the tumor manually; ROIs (shell) were auto-generated by using the shell measurement function and setting shell widths of 0.5-2 mm; and ROI (B) was manually drawn at the normal glandular or fatty tissue. The B/A, B/shell_0.5_, B/shell_1.0,_ B/shell_1.5_ and B/shell_2.0_ was calculated automatically, representing the SR of the internal tumor and that of the peritumoral area from 0.5 to 2 mm width, respectively.

For SWE, the probe touched the skin lightly and the rectangular ROI was adjusted, ensuring that the tumor and sufficient surrounding glandular tissue were included as well as the maximum longitudinal section of the tumor was displayed at the center of the screen. The patient maintained the same position without breathing, when the indicator upper right corner of the screen was nearly green (at least ≥ 4), the satisfactory SWE image was acquired and saved. Similar to the SE index measurement, the border of the tumor was outlined manually and the shell measurement function was used to acquire the elastic modulus of the peritumoral tissues in regions of 0.5-2.0 mm outside the boundary of internal tumors. The maximum, minimum, mean and standard deviation (SD) of elasticity values (kPa) of the tumor and the surrounding tissue were then automatically calculated. The qualitative feature named “stiff rim” sign, was also evaluated in SWE, which is recognized as the typically increased peritumoral stiffness and coded in orange or red as compared with the stiffness in the interior tumor tissues and surrounding tissues [2]. To improve data reproducibility, three measurements were conducted and the average of each variable was calculated. To improve the image quality control, all elastic measurements in our study were conducted by experienced radiologists according to the aforementioned built-in image quality control program in the ultrasound system such as the green control bar in SE and image reliability indicator in SWE to ensure consistent application of pressure during stiffness assessments. Furthermore, the cases with any signs of excessive compression or poor image quality during the measurement process were excluded.

**Appendix A.2 Boruta algorithm for feature selection**

Boruta was an efficient machine learning-based wrapper algorithm for feature selection [3], yielding an all-relevant subset of features that was considered optimal for the prediction outcome. Boruta algorithm uses a wrapper method based on the Random Forest (RF) classifier for feature selection. A “shadow” attribute was created for each feature in the feature pool by shuffling values of the original feature across all patients. Then the shadow attributes are combined with original features for classification using an RF model. The importance of shadow attribute is used as a reference for selecting truly important features, as determined by RF permutation importance measure.

**Appendix A.3 Indications for thyroidectomy and cervical lymph node dissection**

The detailed indications according to the 2015 American Thyroid Association (ATA) guideline for surgical procedures are as follows[4]:

1. For the selection of thyroidectomy:
2. For patients with thyroid cancer >4 cm, or with gross extrathyroidal extension (clinical T4), or clinically apparent metastatic disease to nodes (clinical N1) or distant sites (clinical M1), the initial surgical procedure should include a near-total or total thyroidectomy and gross removal of all primary tumor;
3. For patients with thyroid cancer >1 cm and <4cm without extrathyroidal extension, and without clinical evidence of any lymph node metastases (cN0), the initial surgical procedure can be either a bilateral procedure (neartotal or total thyroidectomy) or a unilateral procedure;
4. For patients with thyroid cancer <1 cm without extrathyroidal extension and cN0, the initial surgical procedure should be a thyroid lobectomy unless there are clear indications to remove the contralateral lobe.
5. For the selection of LND:
6. Therapeutic central-compartment (level VI) neck dissection for patients with clinically involved central nodes should accompany total thyroidectomy to provide clearance of disease from the central neck;
7. Prophylactic central-compartment neck dissection (ipsilateral or bilateral) should be considered in patients with PTC with clinically uninvolved central neck lymph nodes (cN0) who have advanced primary tumors (T3 or T4) or clinically involved lateral neck nodes (cN1b), or if the information will be used to plan further steps in therapy;
8. Thyroidectomy without prophylactic central neck dissection is appropriate for small (T1 or T2), noninvasive, clinically node-negative PTC (cN0);
9. Therapeutic lateral neck compartmental lymph node dissection should be performed for patients with biopsy-proven metastatic lateral cervical lymphadenopathy.

**References:**

1. Itoh A, Ueno E, Tohno E, et al (2006) Breast disease: Clinical application of US elastography for diagnosis. Radiology 239:341–350. https://doi.org/10.1148/radiol.2391041676

2. Zhou J, Zhan W, Chang C, et al (2014) Breast lesions: evaluation with shear wave elastography, with special emphasis on the “stiff rim” sign. Radiology 272:63–72. https://doi.org/10.1148/radiol.14130818

3. Kursa MB, Rudnicki WR (2010) Feature Selection with the Boruta Package. Journal of Statistical Software 36:1–13. https://doi.org/10.18637/jss.v036.i11

4. Haugen BR, Alexander EK, Bible KC, et al (2016) 2015 American Thyroid Association Management Guidelines for Adult Patients with Thyroid Nodules and Differentiated Thyroid Cancer: The American Thyroid Association Guidelines Task Force on Thyroid Nodules and Differentiated Thyroid Cancer. Thyroid 26:1–133. https://doi.org/10.1089/thy.2015.0020


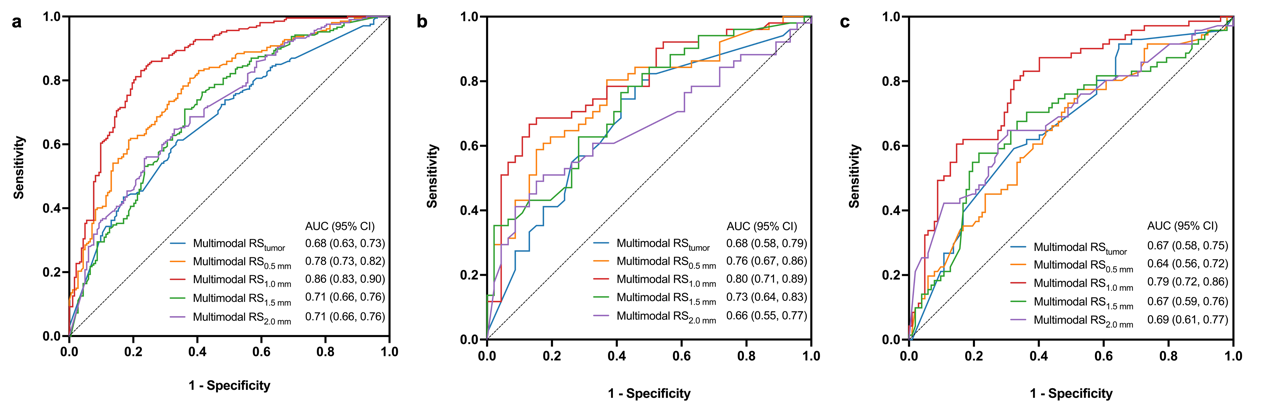


**Figure A.1** ROC curves of the multi-modality radiomics signatures derived from the intratumoral and combined regions with different widths in the training, internal test and external test cohorts.


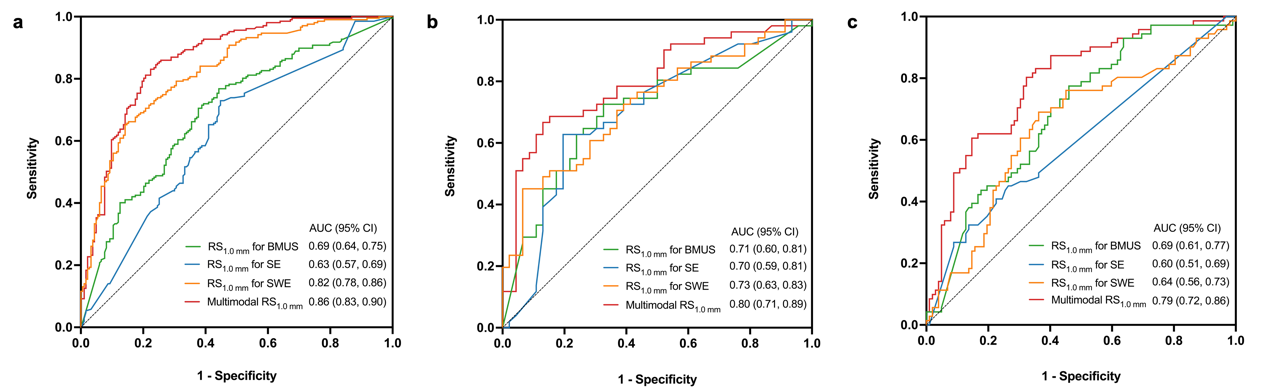


**Figure A.2** ROC curves of single-modality and multi-modality radiomics signatures based on ROI_1.0mm_ in the training, internal test and external test cohorts.


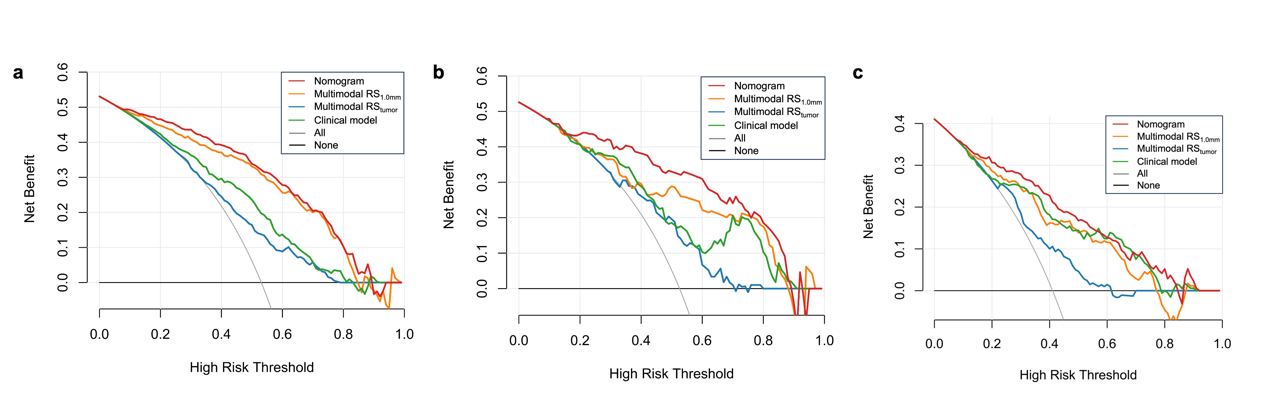


**Figure A.3** Decision curve analysis of different models for predicting cervical LNM in PTC in the (a) training cohort, (b) internal test cohort and (c) external test cohort.


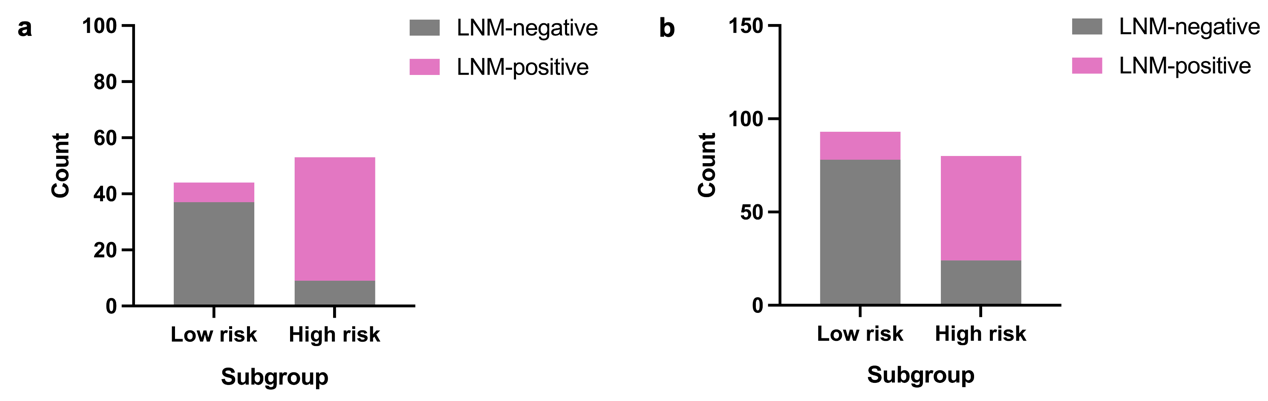


**Figure A.4** The risk-classification performance of the nomogram in the internal test (**a**) and external test cohort (**b**). LNM: lymph node metastasis.


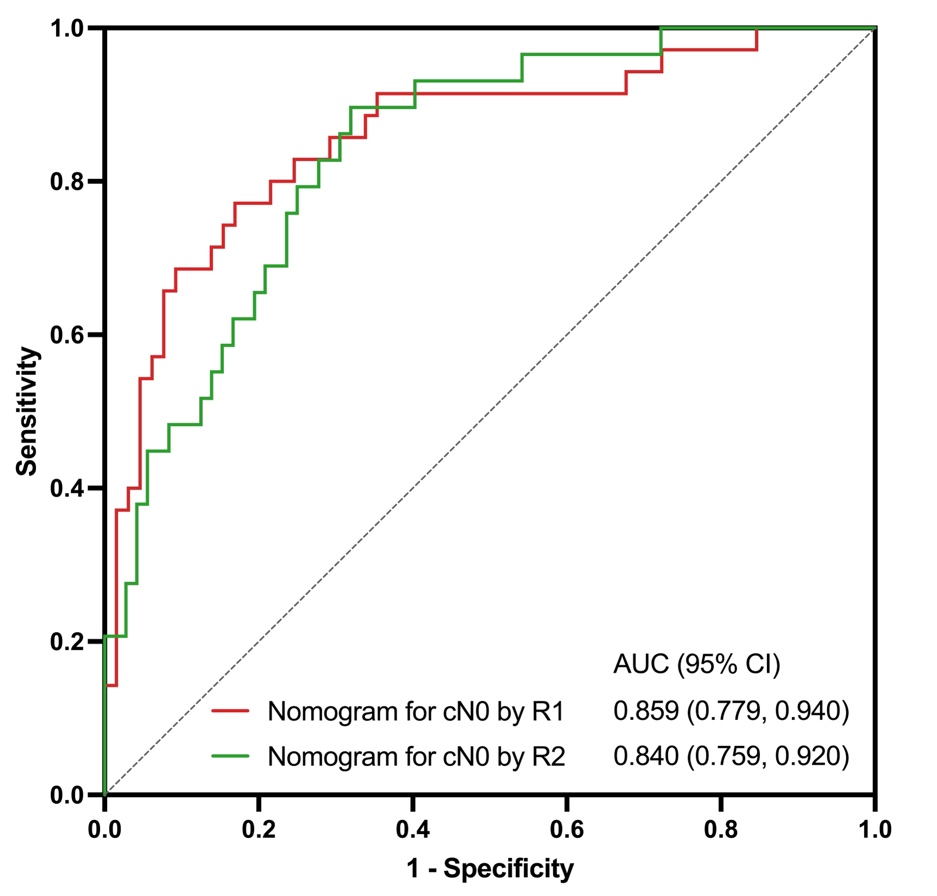


**Figure A.5** ROC curves of the nomogram for the clinically node-negative (cN0) subgroup diagnosed by junior (R1) and senior (R2) radiologists.

**Table A.1** Pathological characteristics of tumors across the three cohorts.

|  | Training cohort  (n = 390) | Internal test cohort  (n = 97) | External test cohort  (n = 173) |
| --- | --- | --- | --- |
| Pathological T stage |  |  |  |
| pT1 | 359 (92.1) | 92 (94.8) | 161 (93.1) |
| pT2 | 23 (5.9) | 2 (2.1) | 9 (5.2) |
| pT3 | 6 (1.5) | 2 (2.1) | 2 (1.2) |
| pT4 | 2 (0.5) | 1 (1.0) | 1 (0.6) |
| Pathological N stage |  |  |  |
| pN0 | 183 (46.9) | 46 (47.4) | 102 (59.0) |
| pN1a | 151 (38.7) | 45 (46.4) | 56 (32.4) |
| pN1b | 56 (14.4) | 6 (6.2) | 15 (8.7) |

Data is shown as numbers of tumors, with percentages in parentheses.

**Table A.2** The univariate and multivariate logistic regression analyses for the independent risk factors of cervical LNM in PTC patients in the training cohort.

| Variables | Univariate logistic regression analysis | | Multivariate logistic regression analysis | |
| --- | --- | --- | --- | --- |
|  | OR (95% CI) | *p*-value | OR (95% CI) | *p*-value |
| Age | 0.969 (0.953, 0.987) | 0.001 | 0.977 (0.958, 0.996) | 0.02 |
| Gender (male) | 0.529 (0.322, 0.869) | 0.01 | 0.504 (0.292, 0.871) | 0.01 |
| Tumor size | 1.099 (1.055, 1.144) | < 0.001 | 1.062 (1.016, 1.110) | 0.01 |
| Shape (aspect ratio ≥ 1) | 0.538 (0.354, 0.817) | 0.004 | NA | NA |
| Microcalcification (present) | 2.257 (1.472, 3.461) | < 0.001 | 1.911 (1.189, 3.072) | 0.01 |
| “Stiff rim” sign (present) | 2.395 (1.552, 3.698) | < 0.001 | 1.703 (1.003, 2.892) | 0.049 |
| E_mean_-tumor | 1.025 (1.006, 1.044) | 0.01 | NA | NA |
| E_max_-tumor | 1.012 (1.007, 1,018) | < 0.001 | NA | NA |
| E_mean_-shell_1.0_ | 1.035 (1.015, 1.055) | < 0.001 | NA | NA |
| E_max_-shell_1.0_ | 1.015 (1.009, 1.021) | < 0.001 | 1.024 (1.010, 1.038) | 0.001 |
| E_sd_-shell_1.0_ | 1.029 (1.004, 1.006) | 0.02 | 0.907 (0.847, 0.972) | 0.01 |

OR: odds ratio; CI: confidence interval

**Table A.3** The number of the retained features after each feature selection procedure of every ROI from each single modality and multi-modality ultrasound images.

| Modality | Input features | Number of features | |
| --- | --- | --- | --- |
|  |  | ICC > 0.75 | Boruta selection method |
|  |  | ROI_tumor_/ROI_0.5 mm_/ROI_1.0 mm_/ROI_1.5 mm_/ROI_2.0 mm_ | ROI_tumor_/ROI_0.5 mm_/ROI_1.0 mm_/ROI_1.5 mm_/ROI_2.0 mm_ |
| BMUS | 944 | 670/670/670/670/670 | 2/2/3/2/4 |
| SE | 944 | 553/553/553/553/553 | 1/3/2/4/0 |
| SWE | 944 | 675/675/675/675/675 | 3/2/4/3/4 |
| BMUS+SE+SWE | 2832 | 1898/1898/1898/1898/1898 | 2/4/6/7/2 |

BMUS: B-mode ultrasound; ICC: Intra-class correlation coefficient; ROI: region of interest; SE: strain elastography; SWE: shear wave elastography.

**Table A.4** The detailed selected features and the corresponding coefficients of RS_1.0mm_ based on multi-modality imaging

| Modality | Feature name | Coefficient |
| --- | --- | --- |
| BMUS | Wavelet-LL_glrlm_GrayLevelNonUniformity | 0.182167873 |
| BMUS | Logarithm_glszm_LargeAreaHighGrayLevelEmphasis | 0.143464908 |
| BMUS | Exponential_glrlm_RunEntropy | 0.212040365 |
| BMUS | Exponential_gldm_SmallDependenceLowGrayLevelEmphasis | 0.145085528 |
| SWE | Wavelet-LH_glcm_Idn | 0.152650803 |
| SWE | Wavelet-LH_glrlm_RunLengthNonUniformityNormalized | 0.164590463 |

BMUS: B-mode ultrasound; SWE: shear wave elastography
